# Dietary Quercetin Intake Is Associated With a Lower Risk of Diabetic Nephropathy in US Adults: Data From NHANES 2007–2008, 2009–2010, and 2017–2018

**DOI:** 10.1002/fsn3.70312

**Published:** 2025-06-12

**Authors:** Fang Liu, Binbin Zhao, Wei Wu, Fang Yang, Ming‐gang Deng, Suqing Wang

**Affiliations:** ^1^ School of Laboratory Medicine Hubei University of Chinese Medicine Wuhan China; ^2^ Hubei Shizhen Laboratory Hubei University of Chinese Medicine Wuhan China; ^3^ Institute of Gerontology Hubei University of Chinese Medicine Wuhan China; ^4^ Engineering Research Center of TCM Protection Technology and New Product Development for the Elderly Brain Health Ministry of Education Wuhan China; ^5^ Affiliated Wuhan Mental Health Center, Tongji Medical College Huazhong University of Science and Technology Wuhan Hubei China; ^6^ School of Nursing Wuhan University Wuhan China

**Keywords:** diabetic nephropathy, NHANES, quercetin

## Abstract

The study aimed to investigate the association between dietary quercetin intake and the risk of diabetic nephropathy (DN) among US adults based on data from the National Health and Nutrition Examination Survey (NHANES) 2007–2008, 2009–2010, and 2017–2018. Dietary quercetin intake was estimated as the mean of two 24‐h dietary recall surveys. DN was defined as a urine albumin‐to‐creatinine ratio greater than 30 mg/g in individuals with diabetes. Basic characteristics between DN and non‐DN groups were compared using the Rao‐Scott *χ* ^2^ test, *t*‐test, and Wilcoxon rank‐sum test. After adjusting for confounding factors, the relationship between dietary quercetin intake and the risk of DN was analyzed using weighted logistic regression. The dose–response relationship between dietary quercetin intake and the risk of DN was analyzed using restricted cubic splines (RCS). Among the 2279 diabetic patients in this study, 645 patients (25.20%) had concurrent DN. Weighted logistic regression analysis showed that, after adjusting for confounding factors, each unit increase in log‐transformed dietary quercetin intake was associated with a 38.10% reduction in the risk of DN (OR: 0.619; 95% CI: 0.457–0.839). The RCS analysis indicated that, after adjusting for confounding factors, there was a linear negative correlation between dietary quercetin intake and the risk of DN (*p* for non‐linearity = 0.059). When dietary quercetin intake was 22.4–65.2 mg/day, the reduction in DN risk was statistically significant. These findings highlighted the potential protective role of dietary quercetin against DN and underscored the importance of dietary interventions as a modifiable strategy for DN prevention among diabetic patients.

## Graphical abstract

The study aimed to investigate the association between dietary quercetin intake and the risk of diabetic nephropathy (DN) among US adults based on data from the National Health and Nutrition Examination Survey (NHANES) 2007–2008, 2009–2010, and 2017–2018. Our findings suggested that dietary quercetin intake was inversely associated with the risk of DN.

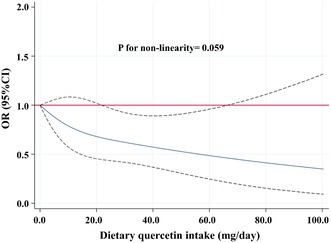

## 1 Introduction

Diabetic nephropathy (DN), characterized by glomerular, tubular, and tubulointerstitial injury, is a major and severe complication of diabetes, contributing significantly to increased risks of cardiovascular disease and all‐cause mortality (Nathan et al. 1993). With the global increase in diabetes prevalence over the past two decades, DN has become the primary cause of end‐stage renal disease (ESRD) (H. Chen et al. 2014). Despite its clinical significance, effective pharmacological interventions to halt or reverse the progression from diabetes to DN remain lacking (K. Yang et al. 2020). Hence, there is an urgent need to seek effective agents to prevent or delay DN progression (Z. Li et al. 2022).

Emerging evidence increasingly highlights the pivotal role of diet in the development and progression of DN, as diet composition could impact the progression of hyperglycemia, hypertension, and dyslipidemia, which are risk factors for DN (Dejenie et al. 2023; Gangadhariah et al. 2015). Several studies showed that high‐fructose (Toyoda et al. 2018) and high‐fat (K. Liu et al. 2019) diets could accelerate the progression of DN. Conversely, consuming a diet rich in fruits and vegetables could reduce the risk of developing DN, primarily due to their high content of flavonoids, vitamins, minerals, and dietary fiber (Moradi et al. 2020; Schwingshackl et al. 2017; Toh et al. 2013).

Flavonoids, a class of polyphenolic compounds, exhibit a range of biological activities, including antioxidant, anti‐inflammatory, anticancer, antimicrobial, and antiviral effects (Niu et al. 2024). The basic structure of flavonoids consists of a C6‐C3‐C6 framework, which includes two benzene rings (A and B rings) and an intermediate three‐carbon chain (C ring) (Niu et al. 2024). Based on the degree of oxidation of the C ring and the type of substituents on the benzene rings, flavonoids could be further classified into flavones, flavonols, isoflavones, flavanones, anthocyanins, and flavanols (Xing et al. 2023). Zhu et al. discovered that supplementation with flavan‐3‐ol could prevent hypoxia‐induced renal injury by upregulating the expression of thioredoxin reductase 1 (Zhu et al. 2022). Shobana et al. and Ademiluyi et al. found that millet and sorghum, which were rich in flavonols, possessed renoprotective properties (Ademiluyi et al. 2014; Shobana et al. 2010). Another animal study demonstrated that supplementation with blackberry juice, which was rich in anthocyanins, could protect the renal system of mice from harmful effects by reducing creatinine levels and regulating catalase activity (de Gomes et al. 2019). Therefore, it is feasible to seek effective substances from the diet, such as flavonoid compounds, for the prevention and treatment of DN.

Quercetin, one of the most common flavonoids found in fruits and vegetables, has broad pharmacological activities, including anti‐inflammatory and antioxidant (Y. Wang et al. 2021). In recent years, some studies have also found that quercetin intervention could protect against DN through in vitro and in vivo experiments. For example, Feng et al. found that quercetin could ameliorate diabetic kidney injury by inhibiting ferroptosis via activating the NFE2‐related factor 2 (Nrf2)/Heme oxygenase‐1 (HO‐1) signaling pathway in DN mice and high glucose (HG)‐incubated renal tubular epithelial cell models (Q. Feng et al. 2023). Liu et al. demonstrated that quercetin ameliorated podocyte apoptosis in db/db mice and HG‐induced mouse podocytes (MPs), and its mechanism was attributed to the inhibition of epidermal growth factor receptor (EGFR) signaling pathway (Y. Liu et al. 2021). Another recent study suggested that quercetin could also inhibit the proliferation of glomerular mesangial cells (MCs) in HG‐treated mouse glomerular MCs and in db/db mice via reactivating the Hippo pathway (Lei et al. 2019). However, the association between dietary quercetin intake and the risk of developing DN in the population remains unclear.

Given the above information, the present study was designed to explore the relationship between dietary quercetin intake and the risk of developing DN using the participants retrieved from the National Health and Nutrition Examination Survey (NHANES).

## 2 Materials and Methods

### 2.1 Study Population

For this cross‐sectional study, we used publicly available data from the 2007–2008, 2009–2010, and 2017–2018 cycles of the NHANES, an ongoing, biennial, nationally representative series of surveys to monitor the health and nutritional status of adults and children in the United States. The methods and data collection procedures behind NHANES were described in detail on the NHANES website: http://www.cdc.gov/nchs/data/nhanes.htm. The protocols for NHANES were approved by the National Center for Health Statistics (NCHS) Research Ethics Review Board, and informed consent was obtained from all participants, available online at https://cdc.gov/nchs/nhanes/irba98.htm. According to 45 CFR Part 46, ethical approval and informed consent were not required for the current study.

**FIGURE 1:**
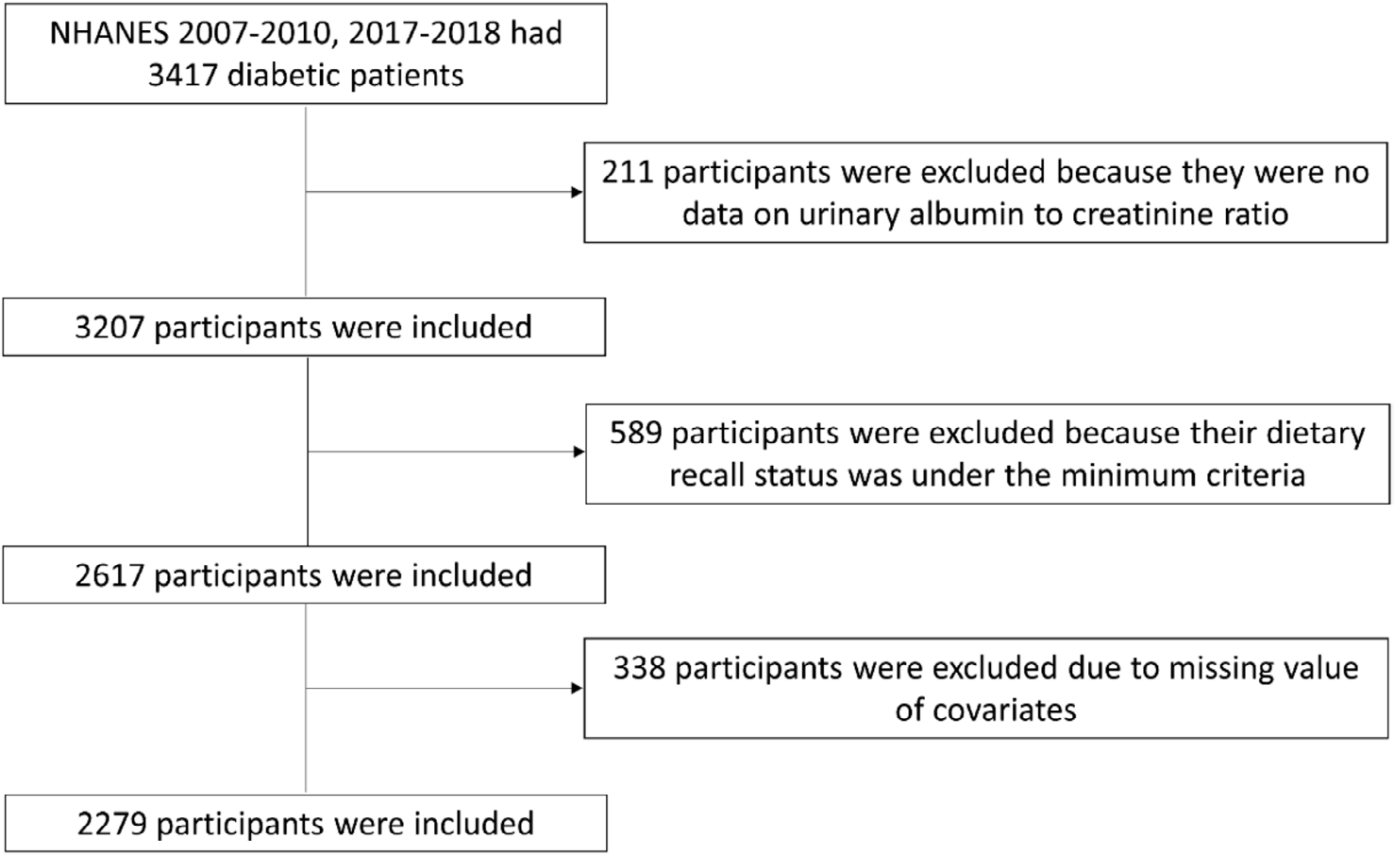
Flowchart of the study population.

Among the 3417 people with diabetes, 211 had no data about urinary albumin to creatinine ratio, 589 had no complete information about diet, and 338 had no complete data about covariates. Finally, 2279 participants were included in the final analysis (Figure 1).

### 2.2 Definition of Diabetes and DN

The diagnostic criteria for diabetes were as follows: (1) self‐reported physician‐diagnosed diabetes; (2) use of insulin or oral hypoglycemic agents; (3) fasting blood glucose (FBG) ≥ 7.0 mmol/L (126 mg/dL); (4) random blood glucose (RBG) ≥ 11.1 mmol/L (200 mg/dL); (5) 2‐h blood glucose in an oral glucose tolerance test (OGTT) ≥ 11.1 mmol/L (200 mg/dL); and (6) hemoglobin A1C (HbA1c) ≥ 6.5%. Meeting any one of these criteria was defined as diabetes (Hanyuda et al. 2020; H. Li et al. 2020; F. Yang et al. 2024). Diabetic patients with a urinary albumin‐to‐creatinine ratio ≥ 30 mg/g were considered to have DN (Bahrampour et al. 2023; Lin et al. 2022). Blood HbA1c, urinary albumin, and urinary creatinine were determined using the A1c G7 HPLC Glycohemoglobin Analyzer, solid‐phase fluorescent immunoassay, and the enzymatic method.

### 2.3 Assessment of Dietary Quercetin Intake

Dietary intake information was assessed through two reliable 24‐h dietary recall interviews, which were conducted in partnership between the U.S. Department of Agriculture (USDA) and the U.S. Department of Health and Human Services (DHHS). Under this partnership, DHHS's National Center for Health Statistics was responsible for the sample design and data collection. USDA's Food Surveys Research Group (FSRG) was responsible for the dietary data collection methodology, maintenance of the databases used to code and process the data, and data review and processing. The first interview was conducted in the mobile examination center (MEC), and the second recall was carried out by telephone 3–10 days later. Most MEC participants (87%) had 2 days of complete and reliable intakes. The release of 2 days of data would permit the estimation of usual (long‐run average) nutrient intakes. Therefore, the mean value of dietary quercetin intake from two 24‐h dietary recall interviews could be adopted as the final measurement of dietary quercetin intake.

### 2.4 Covariates

Based on the previous literature, factors that had been proven to be associated with DN were included in the statistical analysis to control for confounding. Demographic characteristics included sex (male and female), age, race (Non‐Hispanic white, Non‐Hispanic black, Mexican American, and other race) (Tsai et al. 2018), education level (less than a high school diploma, high school graduate/GED, some college/AA degree, and college graduate or more), and marital status (never married, married or living with a partner, and the other). Lifestyle included smoking status (never smoked/former smoker/current smoker), drinking status (non‐drinker/drinker), physical activity level (inactive/low activity/moderate activity/high activity), total dietary energy intake, total dietary protein intake, total dietary carbohydrate intake, and total dietary fat intake. Disease status included body mass index (BMI) [BMI < 25, 25 ≤ BMI < 30, and BMI ≥ 30 kg/m^2^] (Dreimüller et al. 2019), HbA1c, hypertension (hypertensive/non‐hypertensive), and hyperuricemia (hyperuricemic/non‐hyperuricemic).

### 2.5 Statistical Analysis

All analyses were performed using STATA (Version 16.0) and R software (Version 4.1.3). As suggested by the analytic guidelines of NHANES, primary sampling units (SDMVPSU), stratification (SDMVSTRA), and sampling weight (WTDR2D, dietary two‐day sample weight) were incorporated in all analyses to generate nationally representative estimates.

Firstly, categorical variables were expressed as frequencies and weighted percentages, and inter‐group comparisons were performed using the Rao‐Scott *χ* ^2^ test. Continuous variables with a normal distribution were presented as mean ± standard deviation (mean ± SD) and compared between groups using the *t*‐test. Continuous variables with a skewed distribution were expressed as medians and interquartile ranges (P25, P75), and inter‐group comparisons were made using the Wilcoxon rank‐sum test. Three weighted logistic regression models were constructed to explore the relationship between dietary quercetin intake and the risk of developing DN. In these models, dietary quercetin intake was included both as a continuous variable (log‐transformed) and as a categorical variable based on quartiles derived from the original (non‐transformed) intake values. Model 1 was adjusted for sex, age, race, education level, and marital status. Model 2 was adjusted for Model 1 variables plus smoking, drinking, physical activity, total dietary energy intake, total dietary protein intake, total dietary carbohydrate intake, and total dietary fat intake. Model 3 was adjusted for Model 2 variables plus BMI, HbA1c, hypertension, and hyperuricemia.

To test the robustness of the results, two sensitivity analyses were conducted: (1) Age, originally a continuous covariate, was transformed into a categorical variable and included in the logistic regression model, with the following categories: 20–39 years, 40–64 years, and 65 years, and above. (2) To eliminate the confounding effect of hypertension on the results, a logistic regression model was used to analyze the relationship between log‐transformed dietary quercetin intake and the risk of developing DN in a nonhypertensive population.

Subsequently, we conducted stratified analyses to explore the impact of different population characteristics on the relationship between dietary quercetin intake and the risk of developing DN, as well as the interaction between these characteristics and dietary quercetin intake on DN risk. The stratified analyses were performed according to gender (male/female), age (20–39 years/40–64 years/≥ 65 years), race (non‐Hispanic white/non‐Hispanic black/Mexican American/other), education level (less than a high school diploma, high school graduate/GED, some college/AA degree, and college graduate or more), marital status (never married, married or living with a partner, and the other), smoking status (never smoked/former smoker/current smoker), drinking status (non‐drinker/drinker), physical activity level (inactive/low activity/moderate activity/high activity), BMI (BMI < 25, 25 ≤ BMI < 30, and BMI ≥ 30 kg/m^2^), hypertension status (hypertensive/non‐hypertensive), and hyperuricemia status (hyperuricemic/non‐hyperuricemic).

And then, restricted cubic spline (RCS) analysis was conducted to explore the dose–response relationship between dietary quercetin intake and the odds ratios (OR) of the risk of developing DN in US adults with three knots located at the 5th, 50th, and 95th percentiles.

Two‐sided *p* values < 0.05 were considered statistically significant differences.

## 3 Results

### 3.1 Different Characteristics Between DN and Non‐DN Populations

A total of 2279 diabetic patients were included, among which 645 patients had DN, accounting for 25.20%, while 1634 patients did not have DN, accounting for 74.80% (Table 1). Significant differences were observed between the DN and non‐DN groups in terms of race, education level, drinking status, physical activity level, BMI, HbA1c%, prevalence of hypertension, prevalence of hyperuricemia, and dietary quercetin intake. Compared to diabetic patients without DN, those with DN were more likely to be Mexican American, have an education level less than a high school diploma, be non‐drinkers, have low physical activity, be obese, have high HbA1c levels, have hypertension, have hyperuricemia, and have lower dietary quercetin intake. Detailed information is presented in Table 1.

**TABLE 1:** Different characteristics between DN and non‐DN populations.

Variables	Is there diabetic nephropathy		*p*
	No (*n* = 1634, 74.80%)	Yes (*n* = 645, 25.20%)	
Sex (*N*, %)			0.253
Male	830 (50.40%)	364 (54.90%)	
Female	804 (49.60%)	281 (45.10%)	
Age	59.13 ± 13.07	60.85 ± 14.04	0.100
Race (*N*, %)			**0.002**
Non‐Hispanic white	709 (68.00%)	240 (57.10%)	
Non‐Hispanic black	369 (12.40%)	158 (16.40%)	
Mexican American	276 (8.20%)	135 (13.20%)	
Other races	280 (11.40%)	112 (13.30%)	
Education level			**0.012**
< High school	237 (7.80%)	128 (14.20%)	
High school graduate/GED	661 (38.10%)	272 (42.10%)	
Some college/AA degree	463 (31.20%)	167 (26.30%)	
College graduate or more	273 (22.90%)	78 (17.50%)	
Marital status			0.262
Never married	132 (7.40%)	58 (8.00%)	
Married or living with a partner	1007 (66.80%)	372 (61.40%)	
Others	495 (25.80%)	215 (30.60%)	
Smoking status			0.098
Never smoked	802 (49.40%)	295 (41.80%)	
Former smoker	257 (15.10%)	116 (17.70%)	
Current smoker	575 (35.50%)	234 (40.50%)	
Drinking status			**0.018**
Non‐drinker	705 (41.60%)	317 (49.20%)	
Drinker	929 (58.40%)	328 (50.80%)	
Physical activity level			**0.010**
Inactive	1045 (59.20%)	473 (71.40%)	
Low activity	249 (16.20%)	72 (12.00%)	
Moderate activity	150 (12.10%)	55 (7.90%)	
High activity	190 (12.50%)	45 (8.70%)	
Total dietary energy intake (kcal/d)	1835.00 (1343.00, 2315.00)	1778.50 (1350.00, 2289.00)	0.793
Total dietary protein intake (g/d)	73.70 (54.85, 95.57)	72.83 (52.58, 93.54)	0.991
Total dietary carbohydrate intake (g/d)	216.01 (156.56, 270.75)	197.79 (152.76, 258.48)	0.887
Total dietary fat intake (g/d)	71.67 (49.68, 98.20)	69.32 (48.62, 92.62)	0.711
BMI			**0.032**
BMI < 25	190 (12.00%)	78 (7.60%)	
25 ≤ BMI < 30	466 (24.80%)	169 (21.50%)	
BMI ≥ 30	978 (63.20%)	398 (70.90%)	
HbA1c	6.83 ± 1.32	7.73 ± 1.98	**< 0.001**
Hypertension			**0.014**
No	348 (23.50%)	93 (15.40%)	
Yes	1286 (76.50%)	552 (84.60%)	
Hyperuricemia			**0.008**
No	1123 (72.20%)	415 (63.20%)	
Yes	511 (27.80%)	230 (36.80%)	
Dietary quercetin intake (mg/d)	8.46 (4.68, 14.70)	7.72 (3.79, 13.61)	**0.037**

*Note:* Categorical variables were presented as the unweighted count (with the weighted percentage), while continuous variables were presented as the weighted mean ± standard deviation or median and interquartile range (percentiles P25 and P75). The bold values indicate statistically significant differences.

### 3.2 The Association Between Dietary Quercetin Intake and the Risk of Developing DN

Dietary quercetin intake was log‐transformed and analyzed as a continuous variable using three weighted logistic regressions. The results showed that in Model 1, for each unit increase in the log‐transformed dietary quercetin intake, the risk of DN decreased by 39.50% (OR: 0.605; 95% CI: 0.414–0.886). In Models 2 and 3, for each unit increase in the log‐transformed dietary quercetin intake, the risk of DN decreased by 39.60% (OR: 0.604; 95% CI: 0.436–0.836) and 38.10% (OR: 0.619; 95% CI: 0.457–0.839), respectively (Table 2). Additionally, although no significant linear trend across quartiles was observed and none of the associations reached statistical significance, weighted logistic regression analyses showed that the ORs for DN decreased progressively from Q2 to Q4 compared to Q1 (Table 2).

**TABLE 2:** The association between dietary quercetin intake and the risk of developing DN.

	Model 1	Model 2	Model 3
Continuous	0.605 (0.414, 0.886)	0.604 (0.436, 0.836)	0.619 (0.457, 0.839)
Q1 (0.000–0.475 mg/d)	ref	ref	ref
Q2 (0.476–8.280 mg/d)	0.858 (0.550, 1.339)	0.890 (0.589, 1.344)	0.851 (0.549, 1.320)
Q3 (8.281–14.455 mg/d)	0.736 (0.517, 1.047)	0.750 (0.554, 1.015)	0.807 (0.593, 1.099)
Q4 (14.456–100.375 mg/d)	0.739 (0.470, 1.161)	0.732 (0.490, 1.094)	0.740 (0.512, 1.071)
*p* for trend	0.108	0.056	0.074

*Note:* Model 1 was adjusted for sex, age, race, education level, and marital status. Model 2 was adjusted for Model 1 variables plus smoking, drinking, physical activity, total dietary energy intake, total dietary protein intake, total dietary carbohydrate intake, and total dietary fat intake. Model 3 was adjusted for Model 2 variables plus BMI, HbA1c, hypertension, and hyperuricemia.

Abbreviations: CI, confidence interval; OR, odds ratio.

### 3.3 Sensitivity Analysis of the Association Between Dietary Quercetin Intake and the Risk of Developing DN

The results of sensitivity analysis 1, which included age as a categorical variable in the model, showed that for each unit increase in log‐transformed dietary quercetin intake, the risk of DN decreased by 36.10% (OR: 0.639; 95% CI: 0.467–0.874) (Table 3). The results of sensitivity analysis 2, conducted in a non‐hypertensive population, showed that for each unit increase in dietary quercetin intake, the risk of DN decreased by 67.00% (OR: 0.330; 95% CI: 0.144–0.757) (Table 3).

**TABLE 3:** Sensitivity analysis of the association between dietary quercetin intake and the risk of developing DN.

	OR (95% CI)	*p*
Sensitivity analysis 1	0.639 (0.467, 0.874)	0.006
Sensitivity analysis 2	0.330 (0.144, 0.757)	0.010

*Note:* Model was adjusted for sex, age, race, education level, marital status, smoking, drinking, physical activity, total dietary energy intake, total dietary protein intake, total dietary carbohydrate intake, total dietary fat intake, BMI, HbA1c, hypertension, and hyperuricemia.

Abbreviations: CI, confidence interval; OR, odds ratio.

### 3.4 Subgroup Analysis of the Association Between Dietary Quercetin Intake and the Risk of Developing DN

Table 4 illustrated the relationship between dietary quercetin intake and the risk of developing DN across different subgroups based on gender, age, race, education, marital status, smoking status, alcohol consumption, physical activity, BMI, hypertension, and hyperuricemia. The subgroup analysis revealed a linear negative correlation between log‐transformed dietary quercetin intake and DN risk in various groups, including males, those aged 40–64 years, non‐Hispanic whites, non‐Hispanic blacks, Mexican Americans, former smokers, current smokers, non‐drinkers, physically inactive individuals, obese individuals, hypertensive patients, non‐hypertensive patients, and non‐hyperuricemic individuals. Interaction analysis showed that none of the subgroup factors significantly affected the correlation between dietary quercetin intake and DN risk (*P‐interaction* > 0.05).

**TABLE 4:** Subgroup analysis of the association between dietary quercetin intake and the risk of developing DN.

	OR (95% CI)	*p*	*P‐interaction*
Sex (*N*, %)			0.980
Male	0.596 (0.388, 0.917)	0.019	
Female	0.650 (0.386, 1.094)	0.103	
Age			0.409
20–39	0.422 (0.072, 2.460)	0.328	
40–64	0.534 (0.326, 0.873)	0.014	
≥ 65	0.833 (0.499, 1.389)	0.476	
Race (*N*, %)			0.643
Non‐Hispanic white	0.618 (0.393, 0.972)	0.038	
Non‐Hispanic black	0.568 (0.346, 0.933)	0.026	
Mexican American	0.378 (0.165, 0.867)	0.023	
Other races	0.639 (0.298, 1.372)	0.245	
Education level			0.897
< High school	0.589 (0.275, 1.263)	0.169	
High school graduate/GED	0.670 (0.402, 1.118)	0.123	
Some college/AA degree	0.592 (0.265, 1.319)	0.194	
College graduate or more	0.701 (0.234, 2.106)	0.519	
Marital status			0.827
Never married	0.657 (0.239, 1.805)	0.407	
Married or living with a partner	0.601 (0.352, 1.026)	0.062	
Others	0.679 (0.429, 1.073)	0.095	
Smoking status			0.148
Never smoked	0.980 (0.656, 1.464)	0.919	
Former smoker	0.332 (0.181, 0.607)	0.001	
Current smoker	0.404 (0.223, 0.734)	0.004	
Drinking status			0.111
Non‐drinker	0.600 (0.395, 0.911)	0.018	
Drinker	0.760 (0.467, 1.237)	0.263	
Physical activity level			0.277
Inactive	0.614 (0.413, 0.912)	0.017	
Low activity	0.333 (0.110, 1.012)	0.052	
Moderate activity	0.562 (0.150, 2.114)	0.386	
High activity	0.537 (0.140, 2.050)	0.355	
BMI			0.923
BMI < 25	0.505 (0.198, 1.288)	0.149	
25 ≤ BMI < 30	0.739 (0.355, 1.539)	0.411	
BMI ≥ 30	0.622 (0.396, 0.979)	0.040	
Hypertension			0.625
No	0.330 (0.144, 0.757)	0.010	
Yes	0.648 (0.465, 0.904)	0.012	
Hyperuricemia			0.436
No	0.596 (0.393, 0.904)	0.016	
Yes	0.742 (0.461, 1.197)	0.215	

*Note:* Model was adjusted for sex, age, race, education level, marital status, smoking, drinking, physical activity, total dietary energy intake, total dietary protein intake, total dietary carbohydrate intake, total dietary fat intake, BMI, HbA1c, hypertension, and hyperuricemia.

Abbreviations: CI, confidence interval; OR, odds ratio.

### 3.5 Dose–Response Relationship Between Dietary Quercetin Intake and the Risk of Developing DN

**FIGURE 2:**
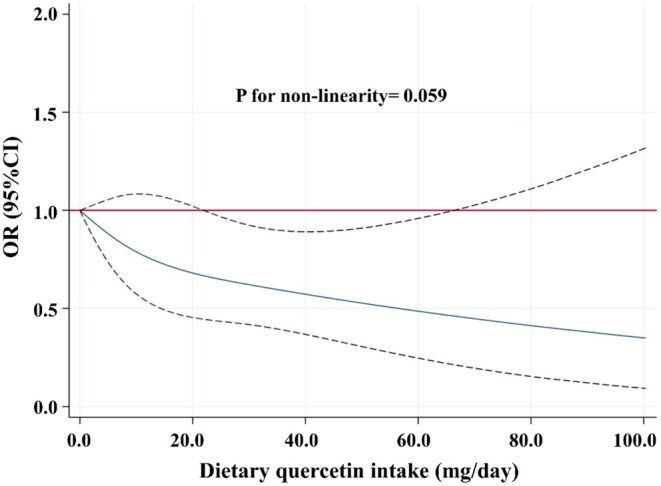
The dose–response relationship between dietary quercetin intake and the risk of developing DN. Point estimates (solid line) and 95% confidence intervals (dotted lines) were calculated by RCS analysis with knots placed at the 5th, 50th, and 95th percentile. The model was adjusted for sex, age, race, education level, marital status, smoking, drinking, physical activity, total dietary energy intake, total dietary protein intake, total dietary carbohydrate intake, total dietary fat intake, BMI, HbA1c, hypertension, and hyperuricemia.

The results of the RCS analysis showed a linear negative correlation between dietary quercetin intake and the risk of developing DN after adjusting for relevant covariates (*p* for non‐linearity = 0.059) (Figure 2). The reduction in DN risk was statistically significant when dietary quercetin intake ranged from 22.4 to 65.2 mg/day.

## 4 Discussion

In recent years, DN has emerged as a significant public health issue threatening human health. The pathogenesis of DN is highly complex, involving genetic factors, inflammatory responses, oxidative stress, and gut microbiota dysbiosis (Holt et al. 2024). Quercetin, one of the most predominant flavonoids in the human diet, has been found in multiple studies to possess various biological activities, including anti‐inflammatory, antioxidant, and antimicrobial effects, playing a crucial role in the prevention and treatment of metabolic diseases such as diabetes, hypertension, and hyperlipidemia (Oyagbemi et al. 2018; Roshanravan et al. 2023). Given the biological effects of quercetin, several recent studies have started exploring its protective role against DN. However, it is noteworthy that this study is the first to investigate the relationship between dietary quercetin intake and the risk of developing DN in a population.

According to the inclusion criteria, 2279 subjects were included in the analysis. When dietary quercetin intake was log‐transformed and analyzed as a continuous variable, weighted logistic regression analysis showed that dietary quercetin intake was inversely associated with the risk of developing DN after adjusting for a series of confounding factors. Additionally, when dietary quercetin intake was categorized into quartiles, the ORs for DN decreased progressively from Q2 to Q4 compared to Q1. However, these associations did not reach statistical significance. This lack of significance might be due to limitations in the exposure classification, which could have resulted in information loss, thus hindering the detection of significant trends. Furthermore, the RCS analysis also found a linear negative correlation between dietary quercetin intake and the risk of developing DN. These findings suggested that increasing dietary quercetin intake might play a significant role in preventing DN in diabetic patients.

Although current studies found an inverse correlation between dietary quercetin intake and the risk of developing DN, the underlying mechanism had not been clarified. However, several possible mechanisms might be applicable to interpret the inverse association between dietary quercetin intake and DN to some extent. Firstly, oxidative stress played an important role in the occurrence and development of DN (M. Chen et al. 2023). One study demonstrated that quercetin could improve renal oxidative stress in diabetic patients by inhibiting nicotinamide adenine dinucleotide phosphate (NADPH) oxidase activity, the main source of kidney ROS (Hu et al. 2022). Secondly, a long‐lasting inflammatory response was generally observed in the pathophysiological process of DN (Shao et al. 2021). Several studies confirmed that quercetin could reduce the production of pro‐inflammatory cytokines such as TNF‐α and IL‐1β in DN (X. Feng et al. 2022; Z. Li et al. 2022). Further exploring the relevant mechanism found that quercetin could inhibit the activation of the nuclear factor‐κB (NF‐κB) signaling pathway and inflammasome of NOD‐like receptor family 3 containing pyrin domain (NLRP3) inflammasome and ultimately inhibit the inflammatory response (P. Chen et al. 2013; C. Wang et al. 2012). Thirdly, accumulating evidence found that abnormal lipid metabolism and renal lipid accumulation play a role in the pathogenesis of DN (M. Yang et al. 2022). Jiang et al. found that quercetin could efficiently alleviate early DN by improving lipid metabolism via the SCAP‐SREBP2‐LDLr signaling pathway (Jiang et al. 2019).

It is noteworthy that although the interaction analysis showed no significant interaction between gender and dietary quercetin intake on the risk of developing DN, subgroup analysis revealed a negative correlation between dietary quercetin intake and DN risk in the male group. However, this association was not observed in the female group. The reasons for this phenomenon could be multifactorial, with one important factor being the significant hormonal differences between men and women, particularly the higher levels of estrogen in women. Chin et al. found that 17β‐estradiol had a protective effect on renal function and histological changes in db/db mice (Chin et al. 2005). Catanuto et al. further discovered that 17β‐estradiol significantly reduced the mRNA expression of transforming growth factor‐β in podocytes and increased the expression of estrogen receptor β, thereby exerting a protective effect against DN (Catanuto et al. 2009). In contrast, the protective effect of quercetin on DN might be much less pronounced than that of estrogen. However, the specific mechanisms underlying the gender differences in the association between dietary quercetin intake and DN risk remain unclear, and further research is needed to explore this issue.

Similarly, the interaction analysis showed that alcohol consumption did not significantly modify the association between dietary quercetin intake and the risk of developing DN. However, subgroup analysis revealed a negative correlation between dietary quercetin intake and DN risk in non‐drinkers, whereas no such association was observed in alcohol consumers. The reasons for this discrepancy might be multifactorial, with one key factor being that excessive alcohol consumption could impair lipid metabolism and hepatic glucose metabolism, leading to abnormal levels of blood lipids and blood glucose, which ultimately accelerate the progression of DN (Lim et al. 2018). Additionally, alcohol is metabolized into various metabolites in the body, which need to be excreted through the kidneys. Chronic and excessive alcohol consumption might increase the renal workload, gradually impairing renal function and promoting or exacerbating the development of DN (D. Li et al. 2019). The side effects caused by alcohol might mask the protective effect of quercetin on DN; however, the specific mechanisms remain to be further elucidated in future studies.

Furthermore, the interaction analysis showed no significant effect of hyperuricemia on the relationship between dietary quercetin intake and the risk of DN. However, subgroup analysis revealed a negative correlation between dietary quercetin intake and DN risk in individuals without hyperuricemia, while no such association was observed in those with hyperuricemia. The reasons for this phenomenon could be multifactorial. First, hyperuricemia is associated with abnormal uric acid metabolism, which might lead to kidney dysfunction and increased DN risk (Wu et al. 2024). Second, elevated uric acid levels could trigger oxidative stress and inflammation, both of which play a critical role in the development of DN (Strazzullo and Puig 2007). Additionally, individuals with hyperuricemia often exhibit unhealthy lifestyles and dietary habits, such as physical inactivity and high‐sugar, high‐fat diets, which might further diminish the protective effect of quercetin. Therefore, future studies should investigate the specific impact of hyperuricemia on the mechanisms of quercetin's action and explore effective interventions to enhance its protective effects in hyperuricemic individuals.

Currently, a limited number of studies have explored the relationship between dietary quercetin intake and the risk of developing diabetes; however, the conclusions of these published studies were not entirely consistent. For example, a cross‐sectional study involving 14,711 participants in China found a negative correlation between dietary quercetin intake and the risk of developing T2DM (Yao et al. 2019). In contrast, a large cohort study including 332,905 middle‐aged and older American women found no association between dietary quercetin intake and the risk of developing T2DM (Song et al. 2005). Although the exact reasons for these contradictory findings are unclear, several factors should be considered: First, genetic background might influence the effect of quercetin on T2DM. Chinese individuals and Western populations have different genetic backgrounds, which may lead to varying susceptibilities to T2DM (Y. Li et al. 2012). Second, studies have shown that the dosage of quercetin could affect its ability to ameliorate T2DM in animal models (T. Yang et al. 2023). The intake of quercetin in the Chinese population was significantly higher than that in Western populations, which might result in the observed negative correlation between quercetin intake and T2DM risk only in the Chinese population (Knekt et al. 2002). Given that genetic background and dietary differences might affect the relationship between dietary quercetin intake and disease risk, it is necessary to further explore the relationship between dietary quercetin intake and the risk of developing DN in other populations.

Our study had several advantages worth pointing out. Firstly, the present study was the first to investigate the relationship between dietary quercetin intake and the risk of developing DN in the population. Secondly, performing sensitivity analyses using multiple methods ensured the robustness of the results. Despite these strengths, our present study has some limitations. Firstly, the assessment of dietary quercetin intake in this study only reflected current intake levels, whereas DN was a disease that developed over a long period, which might introduce bias into the results. Secondly, dietary quercetin intake data were collected through two 24‐h dietary recalls, which might lead to recall bias. Thirdly, this study cannot establish a causal relationship between dietary quercetin intake and the risk of developing DN. Fourthly, the data for this study were sourced from the NHANES database, which caused these findings to be only applicable to the US population.

## 5 Conclusions

Taken together, the present study results demonstrated that dietary quercetin intake was inversely associated with the risk of developing DN in American adults. However, considering the study design of our research, further cell studies, animal studies, large‐scale prospective studies, and clinical trials are needed to confirm these findings.

## Author Contributions

**Fang Liu:** conceptualization (lead), data curation (lead), formal analysis (lead), methodology (supporting), writing – original draft (lead), writing – review and editing (lead). **Binbin Zhao:** conceptualization (equal), funding acquisition (supporting). **Wei Wu:** writing – original draft (equal), writing – review and editing (equal). **Fang Yang:** writing – original draft (equal). **Ming‐gang Deng:** methodology (equal). **Suqing Wang:** writing – original draft (equal), writing – review and editing (lead).

## Ethics Statement

The protocols for NHANES were approved by the National Center for Health Statistics (NCHS) Research Ethics Review Board, according to 45 CFR Part 46, ethics approval was not required for the current study.

## Consent

Informed consent was obtained from all participants available online at https://cdc.gov/nchs/nhanes/irba98.htm. According to 45 CFR Part 46, informed consent was not required for the current study.

## Conflicts of Interest

The authors declare no conflicts of interest.

## Data Availability

The datasets analyzed in this study are publicly available from the NHANES database at https://www.cdc.gov/nchs/nhanes/index.html. All supporting data relevant to the primary findings of this study are presented within the manuscript.
